# Should Super-Selective Intra-Arterial Chemoradiotherapy Be Prioritized over Surgical Resection for Locally Advanced Oral Cavity Cancer?

**DOI:** 10.3390/cancers18030365

**Published:** 2026-01-24

**Authors:** Beng Gwan Teh, Wataru Kobayashi, Kosei Kubota, Shinya Kakehata, Norihiko Narita, Yoshihiro Tamura

**Affiliations:** 1 Department of Oral and Maxillofacial Surgery, Graduate School of Medicine, Hirosaki University, 5-Zaifuchou, Hirosaki 036-8563, Japan; wako@hirosaki-u.ac.jp (W.K.); kkubota@hirosaki-u.ac.jp (K.K.); norihiko@hirosaki-u.ac.jp (N.N.); tam4416@hirosaki-u.ac.jp (Y.T.); 2 Department of Oral and Maxillofacial Surgery, Misawa City Hospital, 65-164 Horiguchi, Misawa 033-0022, Japan; 3 Department of Radiology and Radiation Oncology, Graduate School of Medicine, Hirosaki University, 5-Zaifuchou, Hirosaki 036-8563, Japan; shinya-k@hirosaki-u.ac.jp

**Keywords:** locally advanced oral cavity cancer, oral squamous cell carcinoma, propensity score matching, super-selective intra-arterial chemoradiotherapy, surgical resection, survival rate, quality of life

## Abstract

Background/Objectives: Super-selective intra-arterial chemoradiotherapy (SSIACRT) is an alternatively effective treatment for locally advanced oral cavity cancer although no comparative studies on prognosis between SSIACRT and surgical resection with or without post-operative radiotherapy (S+R) have been reported. This study aimed to compare the 5-year survival rate and Quality of Life (QoL) between S+R and SSIACRT for locally advanced oral cavity cancer. Methods: From a total of 326 patients with stage III and IV oral cavity cancer treated between 2000–2020 at a single institution, 149 patients treated with S+R and SSIACRT were analyzed by using Propensity Score Matching (PSM) method, a pseudo-randomized controlled trial, and the matched cases were retrospectively evaluated. The 5-year survival rate and QoL were evaluated using the Kaplan–Meier method and the University of Washington QoL questionnaire, respectively. Log-rank test and Cox proportional hazards model were used to compare 5-year survival rate and to assess factors affecting survival rates, respectively. Paired *t*-test was used to compare QoL. Results: To compare the 5-year survival rate and QoL between S+R and SSIACRT, 48 and 15 cases were matched after PSM. The 149 cases were further evaluated for covariates affecting survival rates. The 5-year disease-specific survival rate and 5-year crude survival rate were 52.4% and 44.3% for S+R and 71.3%, and 62.9% for SSIACRT, respectively. There was no statistical difference in survival rates between both treatments, based on Log-rank test analysis. Treatment method was the only independent variable that influenced survival rates. SSIACRT showed better statistical difference in QoL evaluation, specifically in appearance, activity, recreation, swallowing, speech, shoulder, taste, mood, and total score. Conclusions: Propensity score-matched analysis demonstrated survival outcomes that were comparable to, and not inferior to, S+R. However, SSIACRT was associated with superior quality-of-life outcomes compared with S+R, as shown by Cox proportional hazards modeling. These findings suggest that SSIACRT is an effective treatment option and, from a quality-of-life perspective, may be considered a preferable approach in the management of locally advanced oral cavity cancer.

## Simple Summary

Treatment of locally advanced oral cavity cancer remains controversial. The standard of care—surgical resection with adjuvant therapies—has not led to significant improvements in overall survival or quality of life, despite advances in radical or invasive treatment and reconstructive techniques. Super-selective intra-arterial chemoradiotherapy (SSIACRT) has emerged as an alternative treatment and has shown outcomes comparable to conventional standard care; however, comparative studies are lacking, and this modality remains less established. To evaluate the efficacy of SSIACRT, we conducted a retrospective study comparing SSIACRT and surgical resection with adjuvant therapies in terms of prognosis and functional outcomes in patients with stage III and IV locally advanced oral cavity cancer. Our findings indicate that SSIACRT is associated with better outcomes, particularly with respect to postoperative quality of life, compared with conventional radical surgical treatment.

## 1. Introduction

Treatment of locally advanced oral cavity cancer has been challenging. Contemporary treatment of multi-modality approaches with surgical resection being the most important treatment procedure is the standard care but the treatment outcomes remain unacceptable [1,2]. Aggressive therapeutic approaches have not led to significant improvements in survival outcomes. Reported 5-year survival rates for locally advanced oral cavity cancer were 50.2% for stage III disease and 16.4% for stage IV disease in 1989, compared with a survival rate of 51.0% for stage III disease and beyond in 2024 [3,4]. Despite continuous advances in surgical techniques, including immediate reconstructive procedures, postoperative disfigurement and functional impairment of the oral cavity remain substantial. Moreover, postoperative chemoradiotherapy is associated with severe toxicity, limited efficacy, and unacceptable adverse events, such as xerostomia and a high risk of osteoradionecrosis [5,6,7]. Super-selective intra-arterial chemoradiotherapy (SSIACRT) is an alternative treatment for locally advanced oral cavity cancer, and its disease-free survival outcomes have been shown to be comparable to those of surgical resection, although overall survival (OS) has not improved significantly [8,9,10,11,12,13]. The efficacy of SSIACRT should have pushed this treatment modal to a more leading role in managing locally advanced oral cavity cancer however, unsignificant OS may have limited the popularity worldwide [14,15,16].

Recently, the JCOG1212 clinical trial, which studied concomitant intra-arterial chemoradiotherapy (RADPLAT), showed effectiveness in improving prognosis and preserving organs in cases of locally advanced maxillary sinus cancer [17]. This has prompted consideration of this treatment model for maxillary sinus cancer as a potential new standard. Comparison of SSIACRT and conventional radical treatment for locally advanced oral cavity cancer is limited. In order to compare the efficacy of the 2 treatments for locally advanced oral cavity cancer, we introduced Propensity Score Matching (PSM) method [18,19] as a pseudo-Randomized Controlled Trial (RCT) to reduce the bias from RCT in this study. The aim of this study is to compare the prognosis and quality of life (QoL) for locally advanced oral cavity cancer after initial radical treatment with mainly surgical resection followed by adjuvant therapies (S+R) and SSIACRT while adjusting the confounding factors through PSM.

## 2. Patients and Methods

### 2.1. Design and Sample

This study is a retrospective analysis of a data from 599 patients with oral cavity cancer treated at the Department of Oral and Maxillofacial Surgery, Hirosaki University Hospital, from 2000 to 2020. The inclusion criteria were as follows: oral squamous cell carcinoma (OSCC) with a TNM classification of stage III to IV, and a World Health Organization (WHO) performance status of 0–1. Tumor progression was classified according to the 7th edition (2010) of the Union for International Cancer Control TNM classification [20]. Patients with distant metastases at the first visit, relapses, or second primary cancers of the head and neck region were excluded from the study. Similar to surgical resection, SSIACRT is an invasive treatment; therefore, a performance status (PS) of 0–1 was required in this study to ensure patient safety. We acknowledge that restricting enrollment to patients with PS 0–1 may have introduced selection bias. Future studies will aim to include patients with a broader range of performance statuses.

### 2.2. Variables

The predictor variable was the type of treatment: S+R or SSIACRT. The primary outcome variable was the 5-year survival rate, while the secondary outcome variable was the QoL score. Covariates included background factors such as age, sex, tumor location, and TN classification.

### 2.3. Data Collection Methods

To adjust for the background factors of both treatment models, a propensity score was calculated using PSM with 1:1 matching ratio. The C-statistic for prognosis and QoL comparison was 0.734 and 0.772, respectively. The caliper was set as 0.04 by multiplying 0.2 by the standard deviation of the propensity score. The study population included unresectable cases, which were allocated to the SSIACRT group. Because resectability cannot be used as a confounding factor in propensity score matching, we performed PSM using age, sex, and TN classification—factors expected to influence prognosis—in order to achieve the greatest possible balance in baseline characteristics. Consequently, the SSIACRT group can be considered disadvantaged compared with the surgery group, as it included more advanced cases.

### 2.4. Treatment Indication

Surgical resection was performed in all operable Stage III and Stage IV oral cavity cancer cases. Patients with multiple lymph node metastases, positive surgical margin, or extra-capsular spread (ECS), as determined by histopathological examination, received 50–66 Gy of postoperative radiotherapy. Since 2003, SSIACRT has been introduced as a radical treatment for locally advanced and inoperable oral cavity cancer, replacing surgical resection. This approach helps address the potential loss of organs and functional impairment within the oral cavity [21].

### 2.5. SSIACRT

SSIACRT was a combination of radiation therapy (66 to 70 Gy) targeting the primary lesion and the entire cervical region, along with super-selective intra-arterial infusion of chemotherapeutic agents using the Seldinger method via the femoral artery. Chemotherapeutic agents were administered three times, every four weeks. The regimen used consisted of 40 mg/mm^2^ of docetaxel and 80 mg/mm^2^ of nedaplatin (Shionogi & Co., Tokyo, Japan), an analog of cisplatin, in a single administration [12].

### 2.6. Methods of QoL Evaluation

The University of Washington QoL (UWQoL) questionnaire, version 4, was used to evaluate QoL. The UWQoL is a head and neck-specific QoL questionnaire designed for patients who have undergone surgery or SSIACRT. Originally developed by Hassan and Weymuller [22], it consists of 15 questions, including 12 disease-specific items such as pain, appearance, activity, resection, swallowing, chewing, speech, shoulder problems, taste, saliva, mood, and anxiety. Additionally, 3 general items that measure global health-related QoL since the diagnosis, and overall QoL. Each of these domains has between 3 and 6 possible answer choices. The highest level of “normal” function is assigned 100 points, while the lowest level or greatest dysfunction is assigned 0 points. The 12 disease-specific domains contribute equally to the final score, which totals 1200 points. Scores for each domain, as well as total scores, were compared among surgery without radiotherapy, surgery with radiotherapy (S+R) and SSIACRT.

### 2.7. Data Analysis

The Kaplan–Meier method was used to calculate the 5-year disease-specific survival rate and the 5-year crude survival rate for the S+R and SSIACRT groups. The statistical significance between the two groups was assessed using the Log-rank test. The Cox proportional hazards model was applied to analyze data prior to PSM to identify factors affecting prognosis. For the QoL assessment, a paired *t*-test was used. Statistical analysis was performed using SPSS version 28 (IBM Corp., Armonk, NY, USA), and a *p*-value of <0.05 was considered statistically significant.

## 3. Results

**Figure 1:**
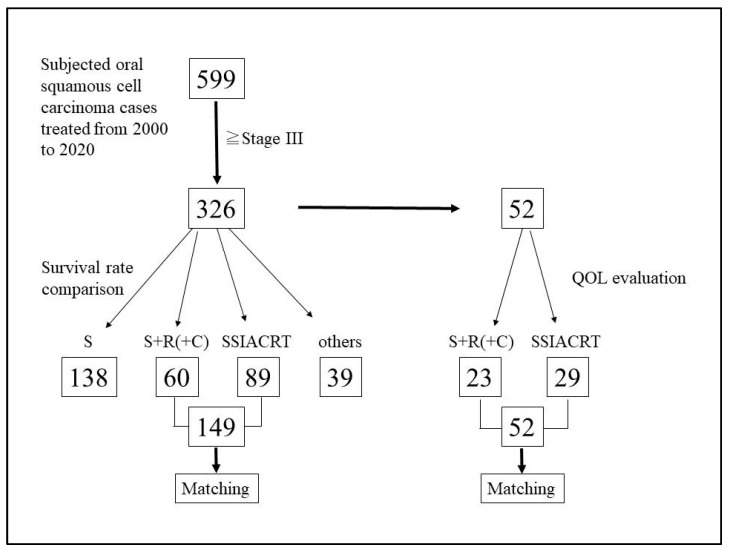
The flow diagram shows the selection of patients for this study. S+R (C): surgical resection with postoperative chemoradiotherapy.

**Figure 2:**
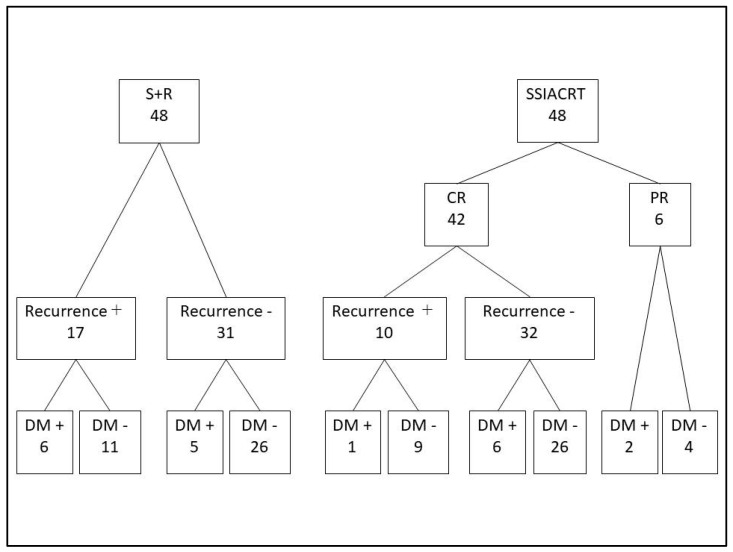
Clinical course of the 96 matched cases. Recurrence was defined as the presence of second primary cancers, late cervical lymph node metastasis, or local recurrence of the primary tumor. DM: distant metastasis.

Of the 599 patients with oral cavity cancer treated at our department between 2000 and 2020, 326 patients diagnosed with stage III or IV disease were included in this study. Among these, 149 patients who were treated with either S+R or SSIACRT were selected for evaluation of the 5-year survival rate. The remaining cases—138 treated with surgical resection alone and 39 treated with chemoradiotherapy or radiotherapy alone—were excluded from the analysis. Figure 1 shows the flow diagram of patients selected for this study, and Table 1 presents the characteristics of the 149 patients treated with S+R and SSIACRT, with 60 patients (38 males and 22 females) in the S+R group and 89 patients (64 males and 25 females) in the SSIACRT group. To compare treatment outcomes, PSM was used, resulting in the selection of 48 patients from each group for the 5-year survival rate assessment (Table 2). Among the 48 patients in the S+R group, 38 underwent initial neck dissection, and of the remaining 10 patients, 7 subsequently received neck dissection due to late cervical lymph node metastases. Eleven patients underwent tumor resection without reconstruction, whereas 34 and 3 patients received free flap and pedicled flap reconstruction, respectively. Postoperative radiotherapy at a dose of 50 Gy was administered to 16 patients, while 32 patients received 60 Gy. Four patients received postoperative chemoradiotherapy with concurrent cisplatin infusion. After PSM, the statistical significance between the S+R and SSIACRT groups was no longer observed. Figure 2 illustrates the flowchart of the clinical course of the matched cases.

**Figure 3:**
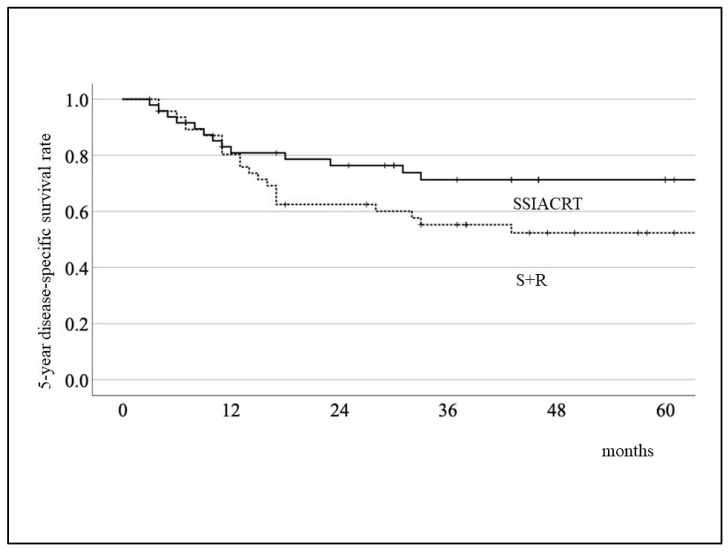
Comparison of 5-year disease-specific survival rate by treatment methods. The 5-year disease-specific survival rates are 52.4% (S+R) and 71.3% (SSIACRT), respectively. Log-rank test: *p* = 0.1.

**Figure 4:**
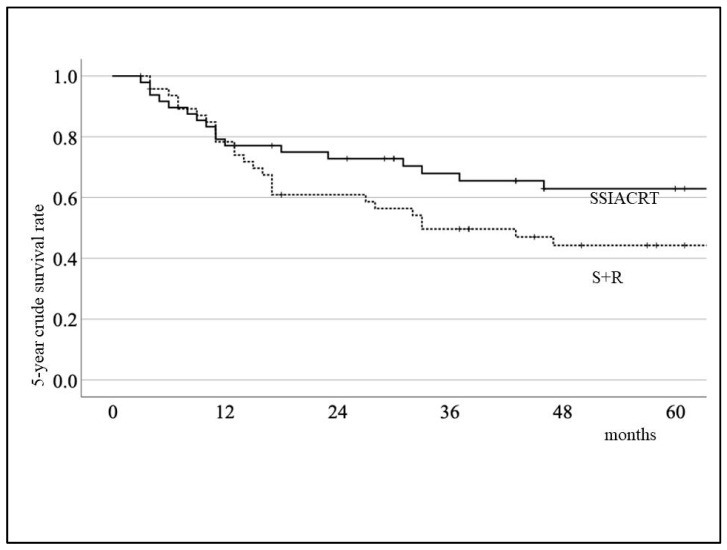
Comparison of 5-year crude survival rate by treatment methods. The 5-year crude survival rates are 44.3% (S+R) and 62.9% (SSAICRT), respectively. Log-rank test: *p* = 0.21.

**Figure 5:**
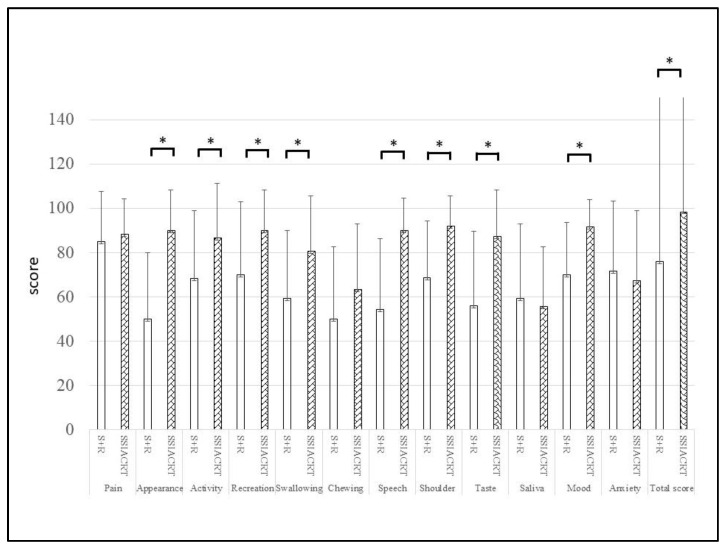
SSIACRT has a better statistical significance in most of the outcomes after the QoL assessment. Paired *t*-test: * *p* < 0.05.

The 5-year disease-specific survival rates for the S+R and SSIACRT groups were 52.4% and 71.3%, respectively, as shown in Figure 3. For the 5-year crude survival rate, the results were 44.3% and 62.9%, respectively (Figure 4). There was no statistical significance in survival rates between the two treatments after evaluation using the Log-rank test. Table 3 shows that the treatment method was the only independent variable affecting survival rates after evaluating the 149 cases prior to PSM using the Cox proportional hazards model. For QoL evaluation, only 52 cases (23 S+R and 29 SSIACRT) from 326 stages III and IV oral cavity cancer cases, with more than 6 months post-treatment, were eligible for selection (Figure 1, Table 4). After PSM, 15 matched cases from each group were evaluated (Table 5). SSIACRT showed significantly better outcomes in appearance, activity, recreation, swallowing, speech, shoulder, taste, mood, and total score, as assessed by paired *t*-test (Figure 5).

## 4. Discussion

Surgical resection, with or without postoperative adjuvant therapy (S+R), has been the standard treatment for oral cavity cancer [1,2]. This treatment provides the best outcomes among the available therapies; however, for locally advanced oral cavity cancer, surgical resection often results in functional loss, leading to poor quality of life (QoL). Chemoradiotherapy (CRT) is an option for curing inoperable locally advanced oral cavity cancer, although the results have been inconclusive. Super-selective intra-arterial chemoradiotherapy (SSIACRT) is an alternative treatment for locally advanced oral cavity cancer and has been the primary curative treatment in our department since 2003. Various reports, including ours, have documented the efficacy of SSIACRT; however, standard care for locally advanced oral cavity cancer remains surgical resection. To determine the superiority of S+R and SSIACRT, we compared and evaluated both treatments in this study, focusing on the 5-year survival rate and functional outcomes. As a result, we confirmed that the SSIACRT group is associated with better outcomes, particularly with respect to postoperative QoL although with the limited sample size after PSM. We will continue our work for future studies with larger sample size and hope our results may lead to a multi-center study.

RCTs theoretically share similar background factors because they randomly determine whether a study subject will receive an intervention. This eliminates bias, even when analyzed by univariate analysis, making RCTs ideal for comparing two different treatments. However, ethical concerns may arise when selecting an established treatment method that can somewhat predict treatment outcomes. In this study, PSM was introduced as a statistical method to unify the background factors. The probability of receiving SSIACRT was calculated as a propensity score, and by matching the background factors on a one-to-one basis, bias was reduced [18,19]. As a result, the retrospective analysis of the S+R and SSIACRT groups could be evaluated as a pseudo-RCT through the use of PSM.

Recently, a multi-institutional clinical trial of Super-selective Intra-arterial Infusion of Cisplatin and Concomitant Radiation Therapy (RADPLAT) for locally advanced maxillary sinus cancer was conducted in Japan [17]. The result demonstrated that RADPLAT is a promising, non-inferior treatment compared to surgical resection and may become the new standard of care for maxillary sinus cancer. The trial highlighted the challenges in introducing RCTs to compare RADPLAT with standard treatment for locally advanced maxillary sinus cancer, due to significant disfigurement, functional impairment after treatment, and patients’ refusal to undergo complete resection of the orbital contents. We share this perspective for locally advanced oral cavity cancer and, like the RADPLAT trial, concluded that RCTs are theoretically inappropriate for evaluating the effectiveness of S+R and SSIACRT. Therefore, we introduced PSM as an alternative method to compare the superiority of both treatments.

Unlike other head and neck cancers, surgical resection had been defined as the standard treatment for oral cavity cancer due to its operable location, the availability of various reconstructive techniques to address functional loss and physical deformity, and the ability to avoid adverse events such as persistence xerostomia, dysgeusia, and the high frequency of osteoradionecrosis that often develop after postoperative chemoradiotherapy [23]. However, the adverse events associated with S+R are inevitable, as CRT with cisplatin infusion is recommended as an additional postoperative treatment for high-risk oral cavity cancer cases to control local recurrence and improve prognosis. It has been reported that, even with successful organ and functional preservation through CRT, the resulting adverse events can compromise function and ultimately lead to poor QoL [24]. Additionally, evidence-based studies comparing S+R and SSIACRT through RCTs are limited, and no study has ever demonstrated the superiority of CRT over S+R for oral cavity cancer.

Iyer NG published the only study that compared S+R and CRT through RCT evaluation. Of the 119 head and neck cancer cases included, only 32 were within the oral cavity [25]. Their results indicated that the 5-year survival rate for oral cavity cancer was 68% for S+R (13 cases) and 12% for CRT (19 cases), with a statistically significant advantage for S+R. However, in our opinion, the limited number of oral cavity cancer cases should not be considered conclusive evidence to define the standard treatment for oral cavity cancer. Retrospective studies specifically focused on oral cavity cancer are limited, and after reviewing previous reports, many indicated that CRT was selected for inoperable cases. However, due to various background factors, the results varied, with some studies showing better statistical significance for S+R [26,27,28], while others reported no significant difference between the two treatments [29,30,31].

SSIACRT is an alternative treatment for locally advanced oral cavity cancer, and our previously published papers are the only reports comparing the effectiveness of S+R and SSIACRT [13,32]. We have stated that SSIACRT provides a better prognosis and QoL for cancers located in the tongue and oral base, and that organ preservation minimizes functional loss of the oral cavity [13]. In this study, evaluating oral cavity cancers from all locations within the oral cavity, we obtained similar results, indicating that SSIACRT offers a better prognosis and QoL than S+R. Postoperative functional loss from surgical resection will be limited to the extent of hemi-glossectomy, with reconstruction implemented. However, even with the application of ideal reconstructive techniques, cases involving sub-total glossectomy or equivalent procedures will experience profound functional impairment. While it is indisputable that S+R is considered the standard treatment for oral cavity cancer, its implementation for locally advanced oral cavity cancer largely disregards the QoL of oral cancer patients.

We perform SSIACRT for cases that either require surgical resection compatible with or greater than sub-total glossectomy, or for high-risk cases where postoperative CRT is highly anticipated. High-risk factors include multiple cervical lymph node metastases, positive surgical margins, and ECS [33,34], although multiple cervical lymph node metastases have been excluded since 2012 [35]. Adverse events from RT do not typically occur in cases that undergo surgical resection. However, in cases with S+R, the side effects of RT can exacerbate the functional impairment resulting from the surgery. The results of this study indicate that SSIACRT offers a better prognosis and QoL by preserving both the organ and function of the oral cavity, compared to S+R. Furthermore, SSIACRT has an advantage over S+R in terms of salvage treatment, particularly in the context of loco-regional recurrence after initial treatment. Based on the results of the present study, SSIACRT appears to be a promising treatment option for locally advanced oral cavity cancer. However, in our view, establishing SSIACRT as a new standard therapeutic option will require the accumulation of further evidence through large-scale, multicenter studies involving a greater number of patients.

## 5. Limitations of the Study

A common high-risk factor in both the surgical and non-surgical groups was the presence of extranodal extension (ECS); however, confounding factors could not be fully controlled in the SSIACRT group. For cases treated before 2012, multiple cervical lymph node metastases were considered high-risk and were managed with postoperative radiotherapy in the surgical group. After concurrent chemoradiotherapy (CRT) with cisplatin infusion became the standard of care, some patients received postoperative chemoradiotherapy; prior to this transition, all patients were treated with radiotherapy alone without concurrent chemotherapy. The current standard dose of postoperative radiotherapy is 60 Gy; however, a dose of 50 Gy was used in some earlier cases. It should also be noted that advances in surgical techniques and instruments over the past two decades may have influenced treatment outcomes.

In addition to the propensity score-matched (PSM) analysis, multivariate analyses were performed on the pre-matching cohort without applying PSM; however, this approach does not benefit from the bias-reducing advantages of PSM. This study was conducted at a single institution with a limited sample size, and the application of PSM further reduced the number of evaluable cases, as observed in the quality-of-life (QoL) cohort. Due to the poor prognosis of the study population, QoL data were available for only 52 patients, and after PSM, only 15 patients remained evaluable. We plan to continue this work and aim to collaborate with multiple centers in future studies to achieve larger sample sizes and more robust analyses.

## 6. Conclusions

In this study, we found that SSIACRT offers a better prognosis and improved quality of life (QoL) compared to S+R for patients with locally advanced oral cavity cancer. SSIACRT not only preserves both organ function and structure but also offers advantages in salvage treatment, particularly for loco-regional recurrence after initial therapy. These findings suggest that SSIACRT may be considered an effective treatment, especially for patients at high risk of postoperative complications or those requiring extensive resection.

However, confounding factors could not be fully controlled in the SSIACRT group, and the sample size was limited, particularly after propensity score matching. These limitations highlight the preliminary nature of our findings. Lessons learned from this study include the value of careful documentation of treatment parameters, the utility of propensity score matching in retrospective analyses, and the need for cautious interpretation of single-institution data. Future studies should address these limitations through prospective, multicenter trials with larger sample sizes and standardized outcome measures. Additional research is also warranted to validate the long-term impact of SSIACRT on survival, functional outcomes, and quality of life, as well as to identify patient- or tumor-specific factors that may predict the greatest benefit from this approach.

## Figures and Tables

**Table 1:** Characteristics of patients subjected to this study.

		S+R	SSIACRT	*p* Value
		n = 60	n = 89	
Age		66.6 ± 10.5	61.2 ± 12.6	0.17 *
Sex	M	38	64	0.29 †
	F	22	25	
Location	Tongue	15	26	0.33 †
	Lower gingiva	22	18	
	Upper gingiva	8	20	
	Oral base	10	16	
	Buccal mucosa	4	8	
	Others	1	1	
T	2	19	12	0.013 †
	3	11	13	
	4	30	64	
N	0	15	19	0.59 †
	1	7	16	
	2	38	53	
	3	0	1	

*: *t*-test, †: χ^2^-test.

**Table 2:** Patients’ characteristics after propensity score matching for survival rates evaluation.

		S+R	SSIACRT	*p* Value
		n = 48	n = 48	
Age		66.3 ± 10.7	65.2 ± 9.8	0.61 *
Sex	M	32	34	0.66 †
	F	16	14	
Location	Tongue	13	11	0.99 †
	Lower gingiva	15	17	
	Upper gingiva	8	8	
	Oral base	8	8	
	Buccal mucosa	3	3	
	Others	1	1	
T	2	13	12	0.96 †
	3	9	10	
	4	26	26	
N	0	13	13	1 †
	1	6	6	
	2	29	29	
Median of observation period (range)		32.5 m (3–27 m)	46 m (4–205 m)	0.84 †

*: *t*-test, †: χ^2^-test.

**Table 3:** Cox proportional hazard models for factors affecting survival rates.

Variables	*p* Value	HR	95.0% CI of HR
Sex	0.918	1.035	0.534~2.006
Age	0.871	1.002	0.974~1.032
Location	0.58	1.07	0.841~1.361
T classification	0.857	1.038	0.69~1.563
N classification	0.242	1.282	0.846~1.944
Treatment	0.042	0.493	0.249~0.976

HR: Hazard Ratio, CI: Confidence Interval.

**Table 4:** Patients’ characteristics for QoL evaluation.

		S+R	SSIACRT	*p* Value
		n = 23	n = 29	
Age		59.0 ± 13.0	60.6 ± 11.2	0.75 *
Sex	M	16	23	0.52 †
	F	7	6	
Location	Tongue	7	9	0.67 †
	Lower gingiva	8	5	
	Upper gingiva	3	7	
	Oral base	5	7	
	Buccal mucosa	0	1	
T	2	9	4	0.11 †
	3	5	5	
	4	9	20	
N	0	8	9	0.87 †
	1	2	6	
	2	13	14	

*: *t*-test, †: χ^2^-test.

**Table 5:** Patients’ characteristics after propensity score matching for QoL evaluation.

		S+R	SSIACRT	*p* Value
		n = 15	n = 15	
Age		56.8 ± 12.4	56.2 ± 12.8	0.89 *
Sex	M	12	12	1.0 †
	F	3	3	
Location	Tongue	3	6	0.707 †
	Lower gingiva	6	5	
	Upper gingiva	2	1	
	Oral base	4	3	
T	2	4	4	1.0 †
	3	2	3	
	4	9	8	
N	0	6	6	1.0 †
	1	1	1	
	2	8	8	

*: *t*-test, †: χ^2^-test.

## Data Availability

The data in this study are available upon request from the corresponding author, subject to valid justification.
